# Early Operation for Appendicitis

**Published:** 1894-08

**Authors:** 


### Editorial.

### EARLY OPERATION FOR APPENDICITIS.

The announcement of the death of Patti Rosa on the 5th inst., after an operation had been successfully performed for appendicitis some days previously, must strike every surgeon with surprise. It is to be expected that a report of the *post mortem* examination in this case will appear in the public prints. But, in the meantime, it may not be out of place to make a surmise as to the probable cause of this sudden fatal termination. It is known that in case of perforation of the appendix vermiformis there is generally a marked vital prostration immediately, ending in profound collapse. After the ligation of the appendix, if the tissues should be cut through before agglutination of the canal is effected, we shall have the escape of the contents of the cecum in the same manner that fecal matter is discharged through a perforation of the appendix. I am therefore prepared to expect that some such result has ensued in this case, and await the verification of the cause of death with much interest.

The pathological conditions associated with perforation of the appendix imply the presence of solid substances within it, either foreign bodies or fecal concretions, which induce ulceration of its walls, with an outlet for its contents. The septic process which has resulted from the inflammation set up by the mechanical irritant imparts a special poisonous quality to the discharge, and the tissues with which it comes in contact loose their vitality and become necrosed very soon after being exposed to it.

I had occasion to note this fact in the reports made of autopsies in several cases where no operation was undertaken, owing to the fulminating effects of perforation of the appendix, and in one case where the destructive process was so rapid and violent that the ligation and removal of the appendix did not avail to avert a speedy fatal result.

It has been observed recently that a writer who has had considerable experience in treating appendicitis had not encountered any case with foreign bodies or enteroliths in the cavity of the appendix. This class of cases must, of course, run a less serious course and prove more amenable to medical treatment than the cases of perforation from the presence of solid bodies attended with mechanical irritation and ulceration of the walls. The latter can only be successfully treated by surgical procedures with the least possible delay, and as no surgeon can know in advance whether these conditions exist in a given case, I am strongly impressed with the great importance of operating promptly in every case where the diagnosis of appendicitis is clearly established.

I have no doubt that some cases will occur in which the patient may recover without any operation, but on the other hand the delay in using the knife must lead to death in many cases which would have been saved by the timely resort to a surgical operation. The great difficulty of deciding in advance which sort of case we are dealing with warrants surgical interference in all cases.

It has recently been a vexed question to decide when an operation is called for in a case supposed to be appendicitis, and it may be laid down as a rule to guide us that upon recognizing the signs of local inflammation with constitutional disturbance, the operation is demanded.

Appendicitis has received so much attention from the profession of late that a successful operation after an established diagnosis in a case of this kind should be recorded. A young man came under the care of Dr. J. S. Todd July 21st, having symptoms indicative of appendicitis, and the writer of this was called in consultation by him July 23d and concurred in his diagnosis. It was determined to watch the developments, and on July 24th it was agreed that the progressive local trouble and constitutional disturbance demanded immediate interference. The operation was accordingly made by the writer with the assistance of Drs. Todd, H. F. Harris and J. McF. Gaston, Jr. An incision four inches in length, extending perpendicularly downward from the level of the umbilicus on the outer border of the right rectus muscle, exposed the cecum, and the appendix was found by careful manipulation at its most dependent part. It was ligated off from the cecal attachment and excised, revealing a small perforation and containing three distinct fecal concretions or enteroliths. Suppuration had already appeared at the lower portion of the incision, which was removed by sponging, and a thick layer of iodoform gauze was packed into the upper portion of the incision temporarily to prevent any extension of the contamination upwards. The peroxide of hydrogen was used for cleansing the purulent focus, and subsequently free irrigation with hot water was employed for all the adjacent tissues. The space below the cecum was filled with iodoform gauze, extending out at the lower angle, thus securing drainage. The peritoneum was closed in the upper half of the incision with catgut, and the muscles, fascia and skin with iron-dyed silk suture. The whole was covered with iodoform gauze and cotton, secured by a roller bandage.

The patient was kept under the influence of morphine with atropine for twenty-four hours, after which Epsom salts with senna tea was given by the mouth and by enema, using also the hypodermatic tablets of magnesium suphate from time to time until the bowels were freely evacuated. There was great discomfort experienced by the patient for several days, but at the end of a week his functions were performed in a normal manner. The stitches were removed one after another from the skin, and adhesive plaster has been used since for bringing the edges of the wound in apposition, keeping gauze in the lower angle.

Upon the tenth day after the operation the ligature was detached from the stump of the appendix and removed. The patient was allowed for the first time to get upon his feet on the fifteenth day, and everything seems favorable to a speedy recovery on the twenty-second day after the operation. J. McF. G.

---

THIS is a utilitarian age. Men in all callings are discarding the useless, often the ornamental, and are holding fast only to short and quick methods. In the practice of medicine this spirit may go too far, and the tried and true may be cast aside for something whose only claim to recognition is its novelty. We fear that in the matter of therapeutics, the profession has, in many instances, made this mistake, and it might be well for us to pause and look thoughtfully into the question of the medicines we are using. It is no easy matter to sift the good from the bad in the application of remedies to diseased conditions. Medicine, above all arts, should be progressive, but it should also be conservative in its progress.

The chemist in his laboratory is busying himself with the synthetic formation of new hydrocarbons, which are no sooner made than the progressive (?) doctor adds them to his armamentarium, and goes forth prepared to meet and conquer disease. That the new substances are oftentimes highly poisonous and unstable compounds, is not considered; they come forth with the imprimatur of some unknown German chemist, and this suffices. Or the bank account of the manufacturing pharmacist grows low and must be supplemented by the sale of some new and elegant compound, which is forthwith made, a sample sent free to every doctor in the land with a statement of its marvelous efficacy and a request that he try it and report. The physician casts aside a substance of known value, tries the new, with, to say the least, doubtful results, gives the manufacturer the benefit of the doubt, and not infrequently furnishes the desired testimonial, which in turn is sent out with the next installment of samples. How many among us are there who, within the last five years, have not tried in a desultory and unscientific manner, countless remedies now discarded as useless and maybe superseded by some newer claimant for popularity. Examine the shelves of any city druggist and see the number of drugs which he would gladly sell at any price, because the physicians have stopped using them.

With these facts staring us in the face, is it any wonder that opprobrium is brought upon the profession for failures and disagreements.

Let us then hold fast to such remedies as have stood the test of time, and while ever ready to investigate, let the investigation be carefully and thoughtfully made; let us compare results and be slow to arrive at conclusions. Deductions to be valuable must be made, not from isolated facts, but from an abundance of concurring testimony.

Lastly, let us frown down the use of patent and semi-patent preparations by men in the profession who know better and are degrading the high calling of medicine by adopting the tools of the charlatan and quack.

WE see from the daily press that at a recent meeting of the Board of Pharmacy of Georgia, the board called attention to the law forbidding the indiscriminate selling of poisons by non-licensed druggists and others not even connected with a drug store. This is a step in the right direction, and both physician and pharmacist should do all in their power to aid in the strict enforcement of the law.

Suicides are of daily occurrence and death is most frequently the result of some poison; morphine or some form of opium being the drug generally used. A large majority of suicides are the result of a diseased or disordered brain, and it becomes the duty of the law to protect these unfortunate people, and it is the duty of the physician to urge the passage and aid in the enforcement of such laws as look to the health and preservation of the masses. Let the physician then, when called to a case of attempted suicide, make it a rule to discover, if possible, where the poison was obtained, and if he finds that it was sold without proper authority, let him report the matter to an officer and see that it is investigated.

---

IT is with sincere regret that we note the death of Dr. A. B. Miles, of New Orleans. Dr. Miles was House Surgeon of the Charity Hospital and Professor of Surgery in the Medical Department of Tulane University, having been elected only last year to the chair of surgery made vacant by the death of Dr. Logan. Dr. Miles was in the full vigor of young manhood, being only forty-two at the time of his death. He was a fluent lecturer and fine operator, and withal as modest and gentle as a woman.

---

THE Supreme Court at Hartford, Conn., has decided, in a suit brought against the New Briton school board to compel them to admit unvaccinated children to the public schools, that the law giving the school board authority to order all school children vaccinated, and to exclude those not vaccinated from the schools, is constitutional.

